# First-in-child phase I trial of p-STAT3 inhibitor WP1066 in pediatric brain tumor patients

**DOI:** 10.1172/jci.insight.194823

**Published:** 2025-12-11

**Authors:** Robert C. Castellino, Hope Mumme, Andrea Franson, Bing Yu, Hope Robinson, Kavita Dhodapkar, Dolly Aguilera, Matthew Schniederjan, Rohali Keesari, Zhulin He, Manoj Bhasin, Waldemar Priebe, Amy B. Heimberger, Tobey J. MacDonald

**Affiliations:** 1 Aflac Cancer and Blood Disorders Center, Children’s Healthcare of Atlanta, Atlanta, Georgia, USA.; 2 Department of Pediatrics, Emory University School of Medicine, Atlanta, Georgia, USA.; 3 Department of Biomedical Informatics, Emory University, Atlanta, Georgia, USA.; 4 Department of Pediatrics, University of Michigan Medical School, Ann Arbor, Michigan, USA.; 5 Department of Pathology, Children’s Healthcare of Atlanta, Atlanta, Georgia, USA.; 6 Department of Experimental Therapeutics, The University of Texas MD Anderson Cancer Center, Houston, Texas, USA.; 7 Department of Neurological Surgery, Malnati Brain Tumor Institute of the Robert H. Lurie Comprehensive Cancer Center, Feinberg School of Medicine, Northwestern University, Chicago, Illinois, USA.

**Keywords:** Clinical Research, Oncology, Brain cancer, Cancer immunotherapy, Clinical trials

## Abstract

**BACKGROUND:**

WP1066 is an orally bioavailable, small-molecule inhibitor of activated phosphorylated STAT3 (p-STAT3) that has demonstrated preclinical efficacy in pediatric brain tumor models.

**METHODS:**

In a first-in-child, single-center, single-arm 3+3 design phase I clinical trial, 10 patients were treated with WP1066 twice daily, Monday-Wednesday-Friday, for 14 days of each 28-day cycle to determine the maximum tolerated dose/maximum feasible dose of WP1066. Compassionate-use treatment with WP1066 in 3 pediatric patients with H3.3G34R/V-mutant high-grade glioma (HGG) is also described.

**RESULTS:**

There was no significant toxicity, and the maximum feasible dose (MFD) was determined to be 8 mg/kg. Treatment-related adverse events were grade 1–2 (diarrhea and nausea most common); there were no dose-limiting toxicities. Median progression-free and overall survival was 1.8 months and 4.9 months, respectively. One partial response was observed in a patient with pontine glioma. Among the H3.3G34R/V-mutant HGG patients not on study, WP1066 was administered after upfront radiation to one patient for 17 months. At all dose levels tested, WP1066 suppressed p-STAT3 expression by peripheral blood mononuclear cells (PBMCs). Single-cell RNA sequencing analysis of PBMCs demonstrated increased CD4^+^ and CD8^+^ T cells, proinflammatory TNFA signaling, differentiation activity in myeloid cells, and downregulation of Tregs after WP1066 treatment, consistent with systemically inhibited STAT3 activity.

**CONCLUSION:**

WP1066 is safe, has minimal toxicity, and induces antitumor immune responses in pediatric brain tumor patients. Phase II investigation of WP1066 at the MFD in this patient population is warranted.

**TRIAL REGISTRATION:**

ClinicalTrials.gov NCT04334863.

**FUNDING:**

CURE Childhood Cancer and Peach Bowl Inc.

## Abstract 2

Phase I trial of STAT3 inhibitor WP1066 in pediatric patients with recurrent brain tumors was found to be safe and generated anti-tumor immune responses

## Introduction

Pediatric high-grade glioma (pHGG), which includes diffuse midline glioma (DMG) and diffuse intrinsic pontine glioma (DIPG), accounts for the majority of deaths related to pediatric brain tumors (1). DMG and DIPG have a dismal median overall survival (OS) of 9–11 months from diagnosis (2). Focal radiation therapy (RT) has proven benefit, albeit transient, when administered at diagnosis or at recurrence for pHGG, but 5-year OS remains lower than 20% (3). Relapsed medulloblastoma (MB) and ependymoma (EPN) are also typically fatal and have no accepted standard therapy at recurrence (4, 5). Thus, each of the 3 most common pediatric malignant brain tumor entities, pHGG, MB and EPN, is in urgent need of novel therapies.

Signal transducer and activator of transcription 3 (STAT3) is a transcription factor that plays critical roles in immune regulation and oncogenesis and is overexpressed in central nervous system (CNS) tumors, including pHGG, MB, and EPN (5–9). STAT3 promotes tumorigenesis by preventing apoptosis and enhancing proliferation, angiogenesis, invasion, and metastasis (10). Growth factors and cytokines activate STAT3 by phosphorylating the tyrosine 705 residue in the STAT3 transactivation domain (p-STAT3), resulting in STAT3-induced expression of target genes (11). Gliomas have high levels of p-STAT3 relative to normal brain tissue, and p-STAT3 expression correlates with tumor grade and decreased survival (12–15).

In H3K27M- and H3.3G34R/V-mutant pHGGs, p-STAT3 is selectively upregulated, and STAT3 signaling promotes survival of H3.3G34R/V-mutant glioma cells (16, 17). Likewise, *STAT3* knockdown or small-molecule inhibition of STAT3 reduces pHGG stem cell viability and restores expression of the polycomb repressive mark H3K27me3 (18), while *STAT3* downregulation in patient-derived DIPG cells decreases cell viability, migration, and invasion (9). Furthermore, radiation has been shown to increase the expression and activation of STAT3 in gliomas (19–21), and combining radiation with STAT3 inhibition significantly increased monotherapy efficacy in HGG models (22). MBs also have high levels of STAT3 (23), and in sonic hedgehog–type (SHH) MB, we have shown that SHH ligand induces expression and activation of STAT3, which is critical to maintaining SHH signaling, promoting cell viability in vitro, and tumor formation in vivo (24).

STAT3 is also a key regulator of immunosuppression and is therefore considered a potential target for cancer immunotherapy (25). STAT3 signaling is induced in immune cells within the tumor microenvironment (TME) (15, 26), which downregulates antitumor immune responses. Immune cells that return to circulation can thus serve as surrogate markers for the TME, as evidenced by significantly increased p-STAT3 expression in peripheral blood mononuclear cells (PBMCs) in patients with glioblastoma multiforme (GBM) (8). p-STAT3 suppresses activation of macrophages (MPs), inhibits natural killer (NK) cell and neutrophil cellular cytotoxicity, and reduces expression of major histocompatibility complex II, CD80, CD86, and interleukin-12 in dendritic cells, rendering them unable to stimulate T cells or to generate effective antitumor immunity (27, 28). STAT3 activation enhances the suppressive activities of human Tregs by upregulating FOXP3 expression (29), and tumor-associated MPs and microglia become polarized via the STAT3 pathway toward tumor-supportive phenotypes (M2) that contribute to angiogenesis and tumor invasion (30).

WP1066 (Moleculin Inc.), a small-molecule congener of caffeic acid benzyl ester able to cross the blood-brain barrier (BBB), is a potent inhibitor of p-STAT3 (31, 32). Unlike most other STAT3 inhibitors, which suppress specific kinase activity upstream of STAT3, WP1066 suppresses p-STAT3 independently of its method of activation in vitro and in vivo. This results in HGG cell apoptosis by downregulating the anti-apoptotic proteins MCL1, BCLXL, and MYC, while activating pro-apoptotic BAX (33). WP1066 can also inhibit vascular endothelial growth factor (VEGF) production and tumor angiogenesis (34, 35). Multiple studies have demonstrated the therapeutic efficacy of WP1066 in various malignancies, including head and neck (36), pancreatic (37), bladder (38), lymphoma (39), gastric (40), CNS melanoma (41), and glioma (42, 43). In genetically engineered murine (GEM) HGG models treated with WP1066, there was a 55.5% increase in median survival, with an associated inhibition of intratumoral p-STAT3 and MPs (42). We have shown that the mechanism of immunologic antitumor cytotoxicity by WP1066 is mediated by a combination of inhibition of Tregs, upregulation of costimulatory CD80 and CD86 molecules on human microglia, induction of proinflammatory cytokine secretion essential for T effector responses, inhibition of immune suppressive cytokines, and the inducement of impaired CD8^+^ effector T cells to become activated and proliferate (22, 31, 41–44). In both orthotopic and GEM models of H3K27M-mutant DIPG and H3.3G34R/V-mutant HGG, WP1066 treatment significantly extended overall survival (16, 17).

Our group previously reported the results of a first-in-human phase I clinical trial of WP1066 in 8 adult patients with recurrent GBM (NCT01904123) (45). WP1066 was found to be well tolerated with a maximum feasible dose (MFD) of 8 mg/kg. Here, we report the results from our first-in-child phase I clinical trial using an identical dosing schedule of WP1066 to treat pediatric patients with recurrent or progressive high-grade brain tumors refractory to standard treatment.

## Results

### Study enrollment and patient characteristics.

**Figure 1:**
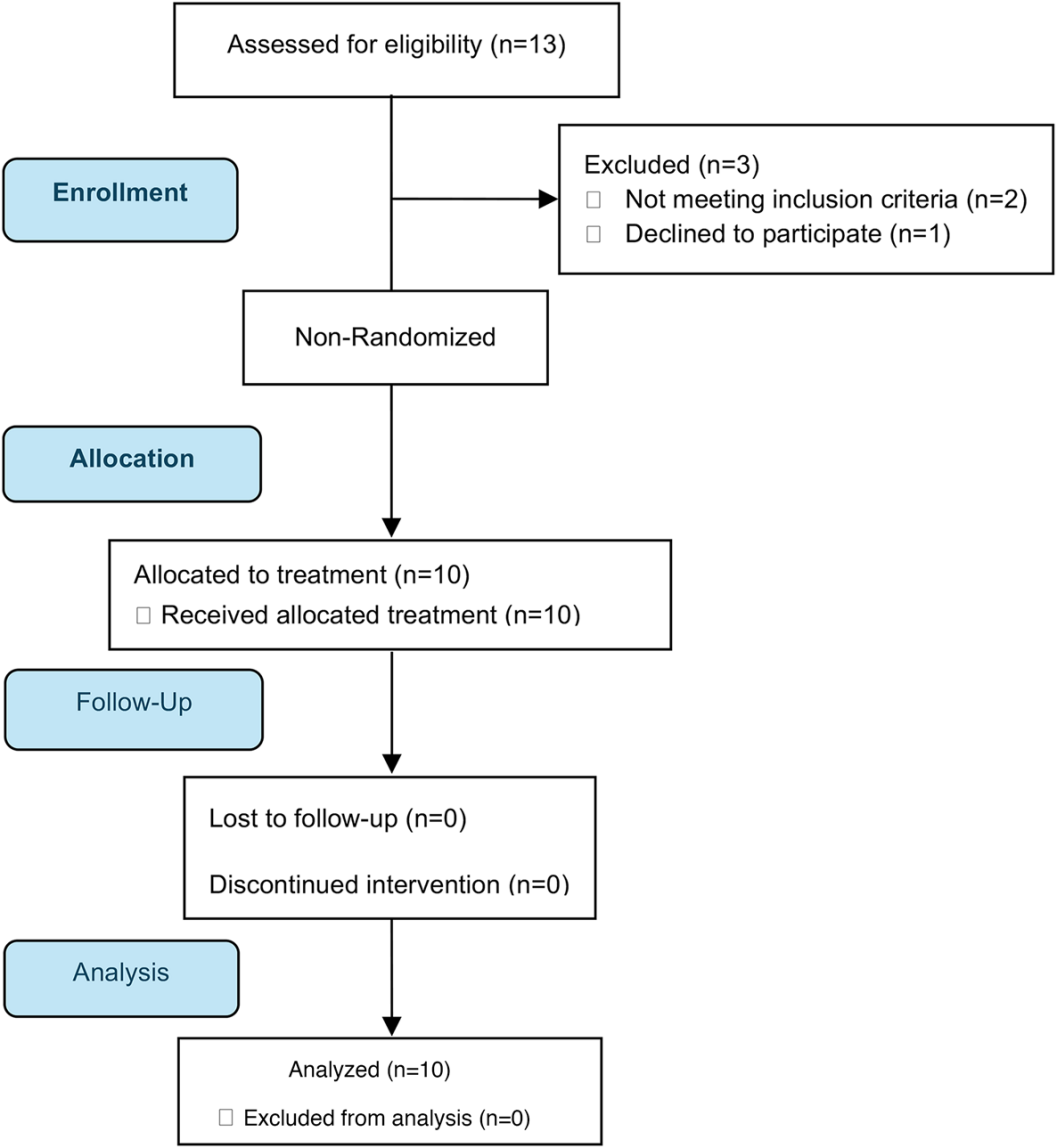
CONSORT diagram of study’s progress from enrollment to final analysis.

The patient flow through the study is shown in the CONSORT diagram (Figure 1). Of 13 patients who gave consent and were screened, 10 patients were eligible and enrolled in the AflacST1901 study between May 1, 2020, and October 26, 2022 (Table 1). Two patients were deemed ineligible because of tumor size or neurologic status at the time of consent. One declined to proceed with trial participation. All patients enrolled in the study had been previously treated with at least standard-of-care therapy, including multi-agent, systemic chemotherapy, and/or radiation therapy. Only 2 patients were receiving corticosteroids at the time of their study treatment (AflacST1901-01 and -010, DIPG and thalamic DMG, respectively). Supplemental Table 1 (supplemental material available online with this article; https://doi.org/10.1172/jci.insight.194823DS1) shows patient demographics and tumor characteristics for 3 young adult patients with supratentorial H3.3G34R/V-mutant HGG who were treated off-study with compassionate-use WP1066 at the University of Michigan C.S. Mott Children’s Hospital.

### Adverse events and maximum tolerated dose/maximum feasible dose.

There were no dose-limiting toxicities (Table 2) and no treatment-related deaths. The most common treatment-related adverse events were grade 1–2 nausea, vomiting, and diarrhea. The most common treatment-related adverse laboratory events were grade 1–2 anemia, white cell count suppression, and elevation of alanine aminotransferase (Table 3). All patients on this pediatric trial reported displeasure with the taste of the liquid formulation of WP1066, and in keeping with the adult phase I trial of WP1066, the maximum tolerated dose/maximum feasible dose (MFD) was determined to be 8 mg/kg. This was also the optimal dose determined for the phase II adult study of WP1066 with upfront radiation. Therefore, the last planned dose level (16 mg/kg) was not investigated in this pediatric trial.

### Patient outcomes.

**Figure 2:**
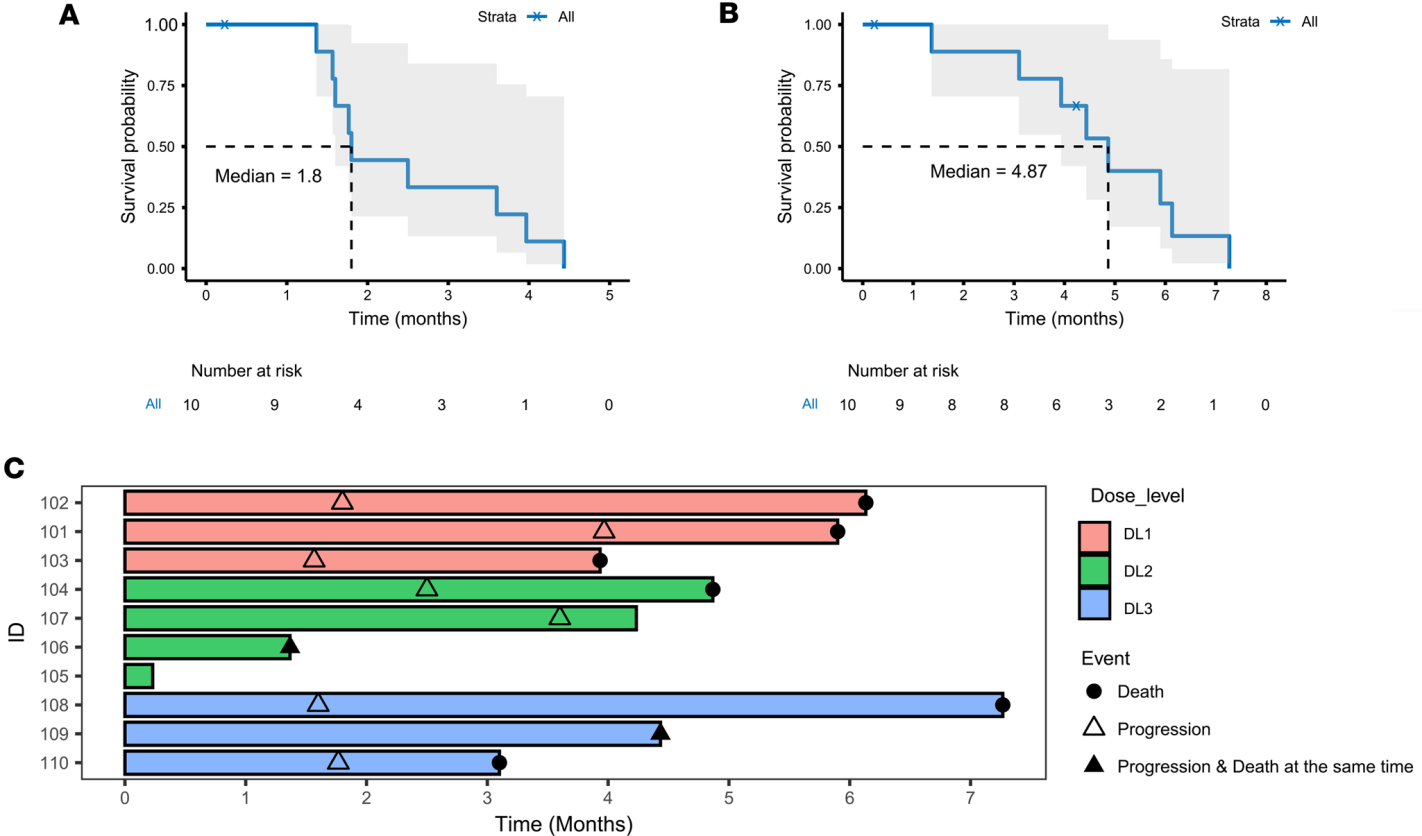
Clinical outcomes of pediatric patients treated with WP1066. (**A** and **B**) Kaplan-Meier analysis is shown for progression-free survival of patients with clinical and radiographic progression based on MRI scans (**A**) and overall survival (OS) of pediatric patients with progressive high-grade brain tumors who were treated on study AflacST1901 with WP1066 (**B**). Shaded area denotes 95% CI. (**C**) Swimmer plot of treatment duration and events for patients on study.

**Figure 3:**
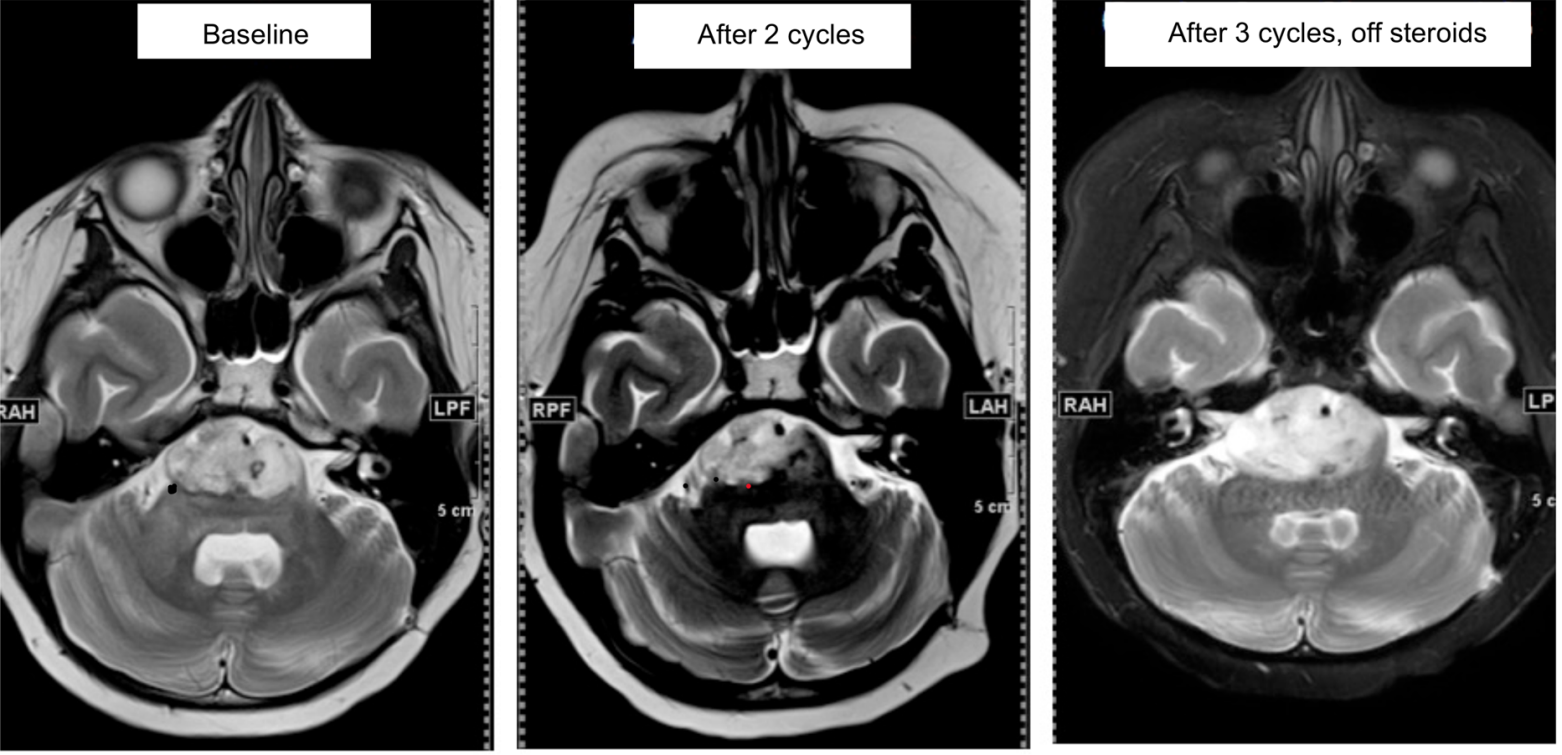
Radiographic tumor response to WP1066 treatment. Serial brain MRIs from a pediatric patient with progressive DIPG (AflacST1901-01) who had completed a 2-week course of palliative focal re-irradiation to the tumor 12 weeks before starting study treatment show 33.8% reduction in tumor volume after 2 cycles of WP1066 treatment compared with baseline MRI obtained 2 days before the start of study treatment (15.48 cm^3^ to 10.25 cm^3^). The patient was successfully tapered off high-dose corticosteroids and improved clinically, from being wheelchair bound to walking with assistance. Subsequent brain MRI performed after 3 cycles of WP1066 while off steroids for 4 weeks (pre–cycle 4) showed a 61.8% increase in tumor volume from baseline (25.05 cm^3^), but no increase in intratumoral blood perfusion, suggesting that the increase in tumor volume was due to treatment-related changes. Because of the increase in tumor volume, the patient was taken off protocol therapy per the study guidelines; however, the patient continued to exhibit clinical improvement followed by stability for an additional 6 months.

The median progression-free survival was 1.8 months, and the median OS was 4.9 months (Figure 2). Of the 10 patients on study with radiographic follow-up, all had eventual radiographic progressive disease and died of disease. However, one patient with progressive DIPG (AflacST1901-01), who had completed a 2-week course of palliative focal re-irradiation more than 4 weeks before enrollment, and had stable tumor size on the baseline pre-study MRI obtained 12 weeks after irradiation and 2 days before starting therapy, subsequently had a 33.8% reduction in maximal tumor volume by MRI by Response Assessment in Neuro-Oncology (RANO) criteria after 2 cycles of treatment with WP1066 (Figure 3). This patient was able to be successfully weaned off high-dose corticosteroids and improved clinically from being wheelchair bound to walking with assistance. A brain MRI after cycle 3 of WP1066 showed a 61.8% increase in tumor size from baseline, but no increase in intratumoral blood perfusion, which was interpreted to be most consistent with treatment-related changes off steroids rather than tumor progression. Per the study protocol, the subject was taken off study treatment at that time because of the increased tumor dimensions; however, the patient continued to exhibit clinical improvement for another 6 months without additional therapy.

For the 3 young adults with hemispheric H3.3G34R/V-mutant HGG who were treated off-study with WP1066 (8 mg/kg) on a compassionate-use basis, the median OS was 4.5 months (Supplemental Figure 1). One patient who was treated with WP1066 after completion of upfront standard radiation and concurrent temozolomide continued adjuvant WP1066 for 17 months before tumor progression.

### STAT3 inhibition and immunologic response to WP1066.

**Figure 4:**
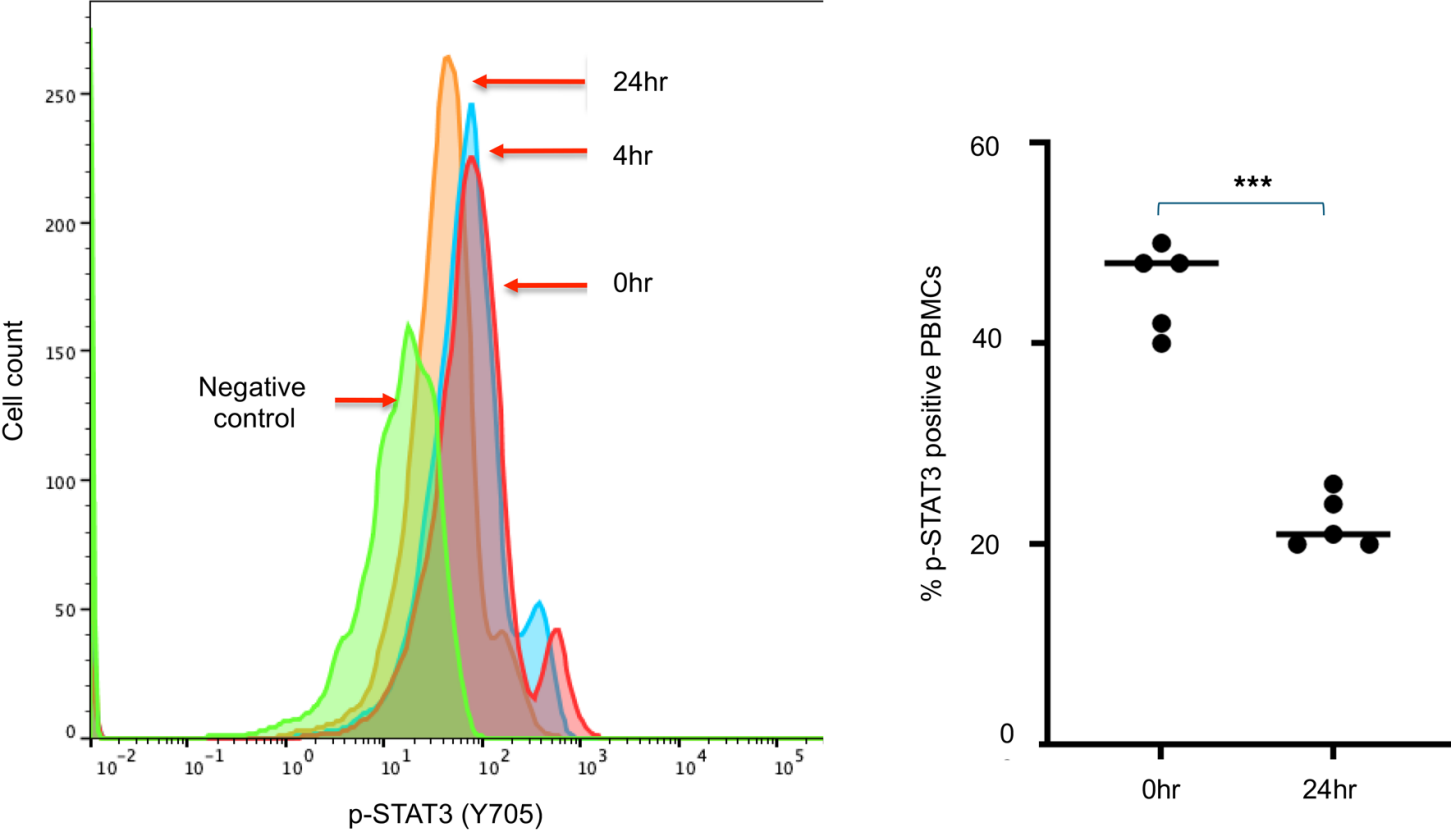
WP1066 suppresses STAT3 activation in PBMCs. Left: Representative flow cytometric analysis of PBMCs from patient AflacST1901-04 during cycle 1, days 1 and 2, demonstrating decrease in phosphorylation (activation) of STAT3 [p-STAT3 (Y705)] at 4 hours and 24 hours after treatment with WP1066 (arrows labeled 4hr and 24hr) compared with baseline pretreatment levels (arrow labeled 0hr) and isotype IgG negative control for p-STAT3. Right: For all patients analyzed (AflacST1901-04, -05, -06, -07, -08), the plot shows a significant reduction in the median percentage of p-STAT3–positive PBMCs from cycle 1, day 1 (C1D1) at hour 0 prior to treatment (0hr) to cycle 1, day 2 (C1D2) at hour 24 after treatment (24hr) with WP1066. Wilcoxon’s rank sum tests were used to compare the percentage of p-STAT3–positive cells from C1D1 0-hour versus C1D2 24-hour time points. Median percentage positive cells is shown by horizontal solid bar; ***P < 0.002. Samples were run in experimental triplicates.

**Figure 5:**
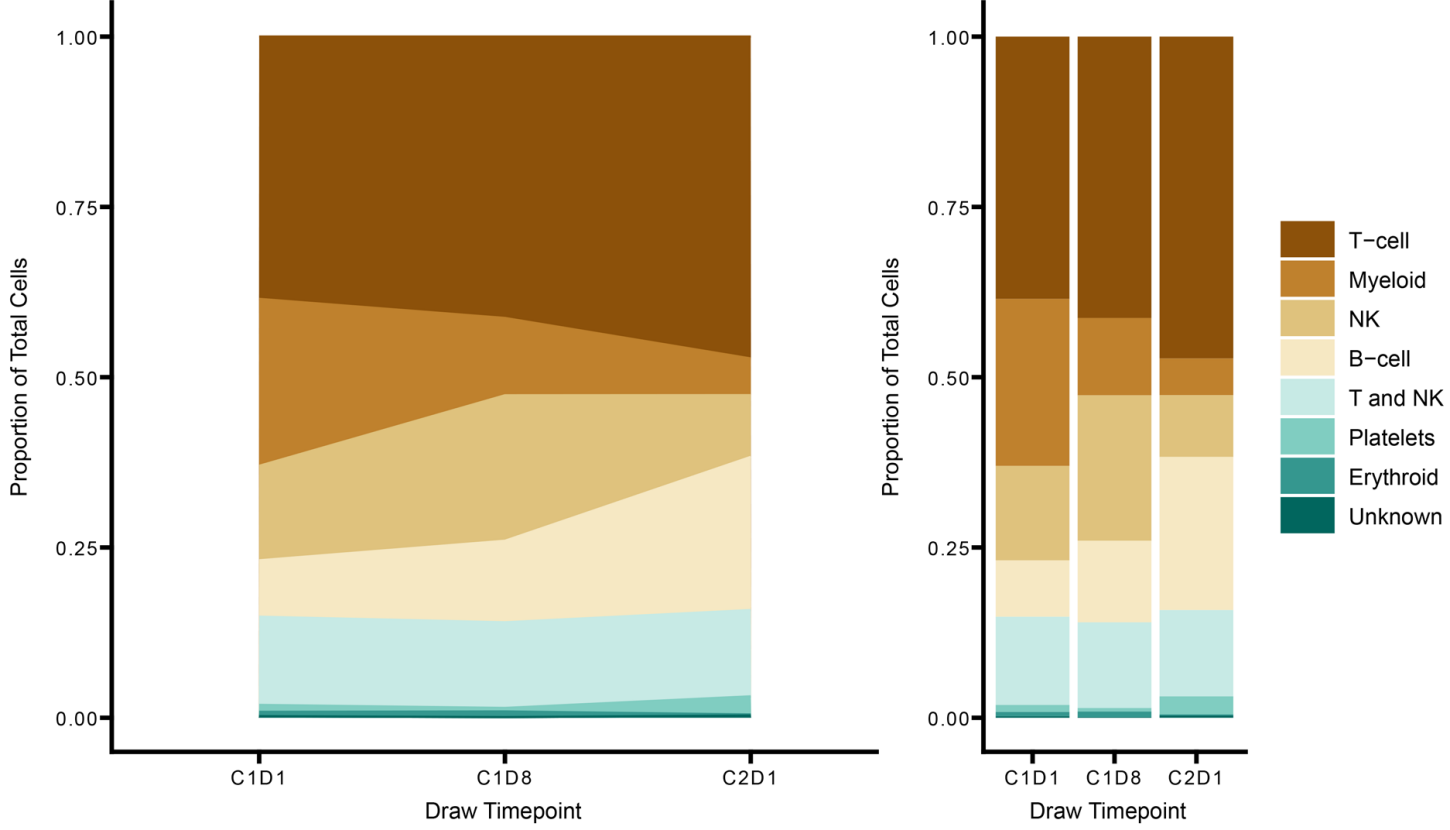
WP1066 alters immune cell compartments in PBMCs. Shown are the proportion of total cells assigned to each cell type in the PBMC samples at each time point: stacked area (left) and bar plots (right). The *y* axis of both plots represents the number of cells labeled as a cell type at the specified time point, divided by the total number of cells for that time point. Shown is an increase in T and B cells from before WP1066 treatment to the start of cycle 2 of treatment with WP1066. PBMCs analyzed correspond to the PBMC samples analyzed by flow cytometry demonstrating suppressed p-STAT3 after WP1066 treatment (AflacST1901-04, -05, -06, -07, -08) and one additional subject (AflacST1901-010) who did not have flow cytometric analysis for PBMC p-STAT3. Only subject AflacST1901-010 received concomitant corticosteroids during treatment, and the dosage of steroids administered remained constant throughout. To identify the proportion of CD4^+^ and CD8^+^ T cells present in the PBMCs, module scores were calculated (Seurat v4) for all T cells and for different T cell subtype signatures from the literature (56). In addition, gene set enrichment analysis was performed to assess the underlying biological mechanisms active in the PBMCs at different time points. The escape package (60) was used to assess the enrichment of different hallmark gene sets (MSigDB).

**Figure 6:**
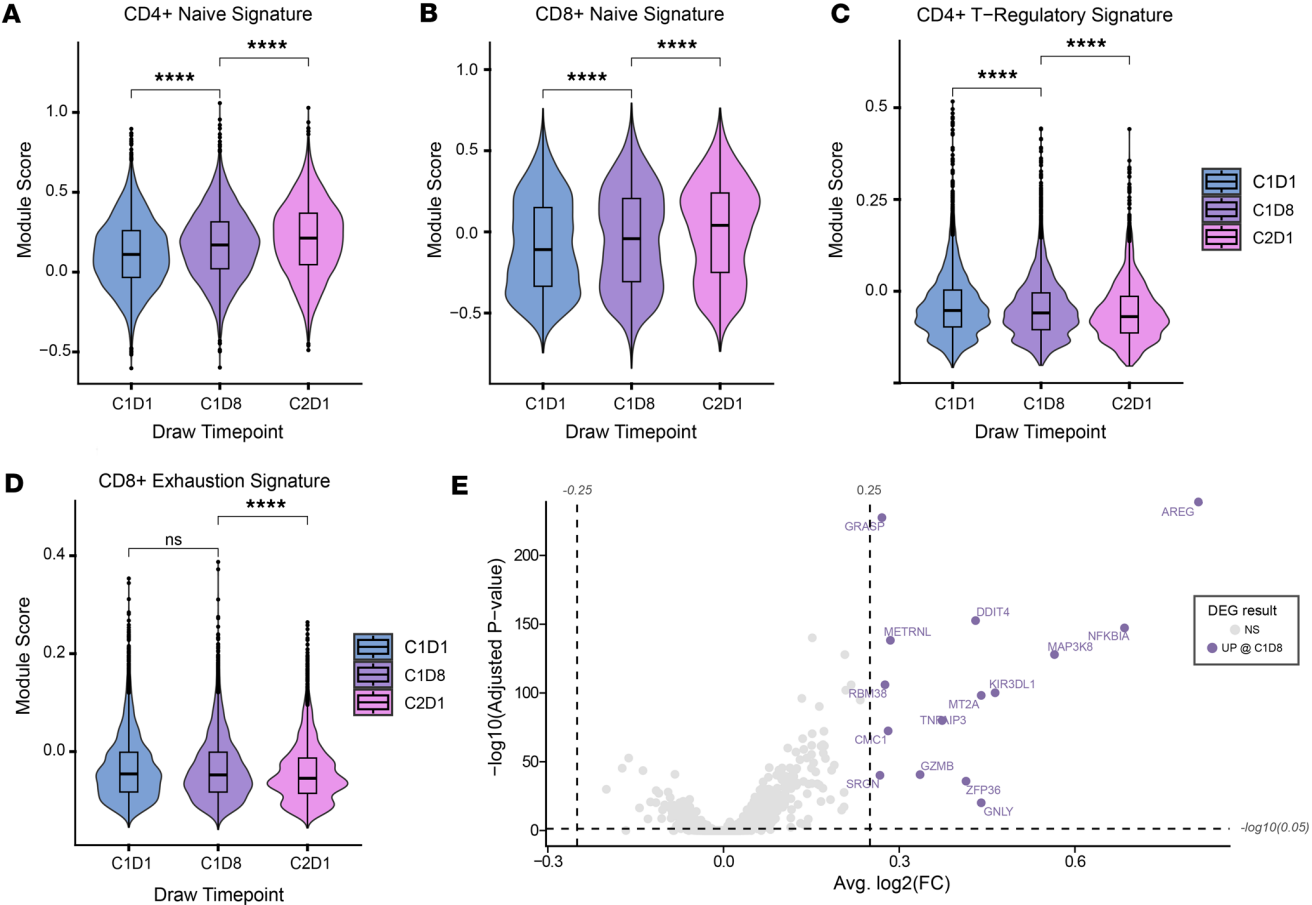
Changes in T cell subsets in response to WP1066. CD4^+^ and CD8^+^ T cell subsets were estimated using phenotype signatures from the literature and assessing the expression of these signatures in T cell subsets in PBMCs. PBMCs analyzed correspond to the PBMC samples analyzed by flow cytometry demonstrating suppressed p-STAT3 after WP1066 treatment (AflacST1901-04, -05, -06, -07, -08) and one additional subject (AflacST1901-010) who did not have flow cytometric analysis for PBMC p-STAT3. Only subject AflacST1901-010 received concomitant corticosteroids during treatment, and the dosage of steroids administered remained constant throughout. Module score function from Seurat was used to represent the aggregate expression of these signatures in the cells. (**A** and **B**) Module scores for CD4^+^ (**A**) and CD8^+^ (**B**) naive signatures are shown for the designated time points. (**C** and **D**) Module scores for Treg signature (**C**) and CD8^+^ T cell exhaustion signature (**D**) are shown. Wilcoxon’s rank sum tests were used to compare module scores for cells from C1D1 versus C1D8 and C1D8 versus C2D1 time points. The resulting *P* values are shown: ****P < 0.0001; ns, not significant, *P* > 0.05. (**E**) Differential expression analysis was performed to identify genes significantly changing in expression in T and NK cells between C1D1 and C1D8 time points. Genes increasing after treatment (UP @ C1D8, purple) were identified based on the adjusted *P* value and average log_2_(fold change) [log2(FC)] results [adjusted *P* value < 0.05, average log2(FC) > 0.25]. DEG, differentially expressed gene.

**Figure 7:**
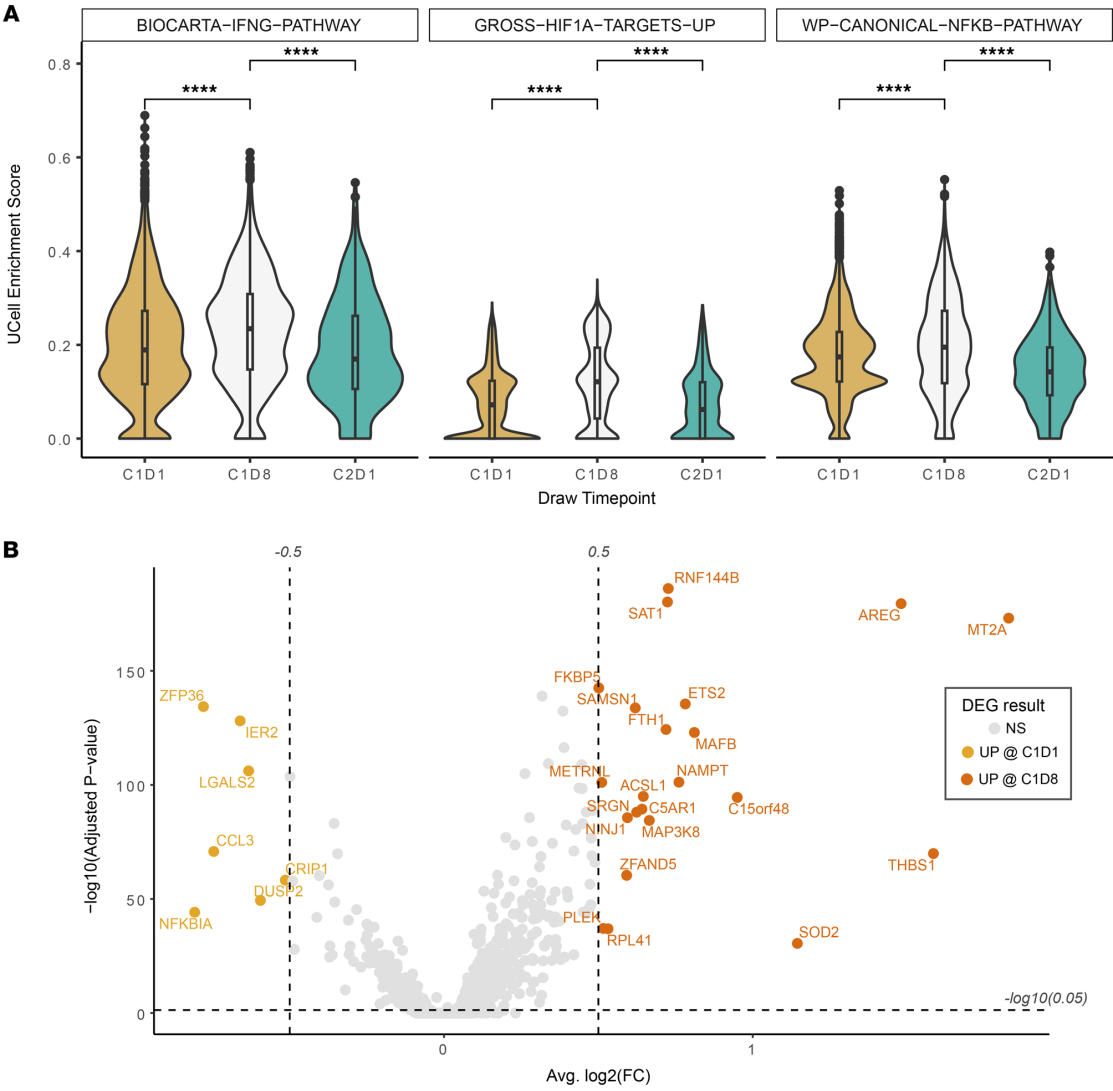
The upregulated inflammatory pathways and markers in myeloid cells with WP1066 treatment. (**A**) Gene set enrichment analysis was performed to calculate the enrichment of the inflammatory pathway gene set in myeloid cells across time points. Gene sets were obtained from the MSigDB collection. Wilcoxon’s rank sum tests were used to compare enrichment scores among cells from C1D1 versus C1D8 and C1D8 versus C2D1 time points. Cell counts are shown in the figure legend. ****P < 0.0001. (**B**) Differential expression analysis was performed to identify genes significantly changing in expression in myeloid cells between C1D1 and C1D8 time points. Genes increasing after treatment (UP @ C1D8, orange) and decreasing after treatment (UP @ C1D1, gold) were identified based on the adjusted *P* value and average log_2_(fold change) [log2(FC)] results [adjusted *P* value < 0.05, |average log2(FC)| > 0.5]. DEG, differentially expressed gene.

Flow cytometric analysis of PBMCs for phosphorylated STAT3 (p-STAT3), which indicates STAT3 activation, demonstrated a significant reduction in p-STAT3 within 24 hours of WP1066 treatment in all patients analyzed (*n* = 5, AflacST1901-04, -05, -06, -07, and -08), confirming on-target activity of WP1066 in these pediatric patients (Figure 4). Analyses of p-STAT3 in PBMCs by day 8 (D8) of therapy showed that the p-STAT3 signal had reverted to baseline at cycle 1, day 1 (C1D1), hour 0, and the subsequent decreases in p-STAT3 following treatment for C1D8 at 4 hours and 24 hours were virtually identical to that at C1D1 at these same time points (data not shown), indicating that there is not a prolonged or cumulative effect on p-STAT3 with each successive dose of WP1066. Analysis by scRNA-seq of 17 PBMC samples from the 5 corresponding study patients with PBMC p-STAT3 flow cytometric analysis, plus 1 additional subject (AflacST1901-010), demonstrated an overall increase in T and B cells before initiation of treatment to the start of cycle 2 on treatment (Figure 5, Supplemental Table 2, and Supplemental Figure 2). Of the patients analyzed, only subject AflacST1901-010 had received concurrent corticosteroids during protocol therapy, and the dosage of steroids had been stable since 1 week before the start of WP1066 therapy, indicating that the changes observed in immune cells with treatment were not related to concomitant changes in the dosage of steroids administered. scRNA-seq further showed an increase of gene signatures consistent with an increase in naive CD4^+^ (Figure 6A) and CD8^+^ T cells (Figure 6B) and a decrease in the Treg signature within the CD4^+^ T cell compartment (Figure 6C) consistent with reduced STAT3 activation. Meanwhile, the gene signature of exhausted CD8^+^ T cells decreased over that time (Figure 6D). Gene set enrichment analysis of the PBMCs revealed reduced tumor necrosis factor-α (TNFA) signaling, especially in CD8^+^ T cells. In addition, differential expression analysis between T and NK cells before (C1, D1) and after treatment (C1, D8) revealed increased expression of cytotoxicity-associated genes (*GZMK*, *GNLY*) (Figure 6E). Cumulatively, this demonstrates peripheral antitumor immune system changes in response to treatment with WP1066. CD8^+^ T cells exhibited upregulation of gene sets associated with DNA repair, the G_2_/M cell cycle checkpoint, and apoptosis from before treatment to C1D8 of WP1066 treatment (Supplemental Figure 3). STAT3 activation is known to reduce immune cytotoxicity effector functions, and WP1066 treatment increased TNFA signaling in the PBMCs from before treatment to C1D8 of WP1066 treatment, suggesting increased cytotoxic immune cell activity. STAT3 activation is also known to suppress the activation of MPs and limit inflammatory responses in the TME. WP1066 treatment resulted in the upregulation of genes associated with regulation of inflammatory pathways (IFNG, HIF1A targets, NFKB) in myeloid cells from before treatment to C1D8 of WP1066 therapy (Figure 7A and Supplemental Figure 4). In addition, inflammation regulation (*MAP3K8*, *NAMPT*) (46, 47), stress (*FKBP5*) (48), and differentiation-related (*MAFB*) (49) genes were identified as significantly increasing in expression from before treatment to C1D8 (Figure 7B). Overall, this indicates a reduction in systemic immunosuppression within 8 days of treatment with WP1066, indicating that there are longer-term impacts on the immune response as a result of the transient decreases in p-STAT3 with each WP1066 dosing interval.

## Discussion

This phase I clinical trial was a first-in-child study of the orally administered BBB-penetrant p-STAT3 inhibitor WP1066. Here, we identified an MFD of 8 mg/kg on a Monday-Wednesday-Friday schedule for the first 14 days of each 28-day cycle in children with refractory brain tumors. Dose escalation beyond 8 mg/kg was stopped because patients expressed oral displeasure at this dose, and because 8 mg/kg was determined to be the phase II adult dose for WP1066 in combination with radiation therapy (RT) based on preclinical testing showing that allometric 8 mg/kg is optimal for promoting an antitumor immune response in mouse GBM models (22). It is worth noting that premedication with a viscous lidocaine mouth spray and antiemetics increased the oral tolerability of WP1066. Otherwise, the drug was well tolerated with minimal toxicity, confirming that administration of WP1066 in children with brain tumors is safe and feasible.

The median progression-free survival and overall survival, 1.8 and 4.9 months, respectively, are similar to the survival rates reported in the adult GBM phase I trial (45). Most notable was the one patient with DIPG who, after being taken off study for progression, continued to exhibit significant improvement in physical function for another 6 months off steroids and without further therapy, suggesting that the initial increase in tumor size may have been due to treatment-related pseudo-progression. Based on historical data, the timing and duration of this patient’s radiographic and clinical improvement with WP1066 treatment, which started more than 4 weeks after palliative re-RT, could be considered atypical for the clinical course following re-RT alone in a steroid-dependent patient with progressed DIPG (50). Likewise, among the H3.3G34R/V-mutant HGG patients treated with compassionate-use WP1066, one remained free of tumor progression for 17 months while on WP1066 treatment after completion of upfront RT. This clinical course is also atypical for H3.3G34R/V-mutant HGG, which historically has a median progression-free survival of about 10 months (51). No other patients reported here received RT within 4–6 weeks prior to starting WP1066. Together, these results suggest that WP1066 may have clinical benefit after RT in select brain tumor patients (i.e., STAT3-dependent tumors such as DIPG and H3.3G34R/V-mutant HGG) (9, 16, 17). Indeed, in preclinical models of GBM, it was not until RT was used in combination with WP1066 that a therapeutic response resulting in long-term survival and significantly enhanced median survival was observed (22). In that murine model, the efficacy of combining RT with WP1066 appeared to be due to RT-induced immune stimulation rather than RT-mediated cell killing, as this effect was lost in immune-incompetent mice.

PBMC p-STAT3 is upregulated in adult HGG patients (8) and thus could serve as a potential surrogate marker for STAT3 activity in the TME. In the adult GBM phase I trial, patients demonstrated suppression of PBMC p-STAT3 expression by flow cytometry, even at the lowest WP1066 dose tested (1 mg/kg), as early as 172 hours after treatment (45). In comparison, flow cytometric analysis of patient PBMCs in our trial demonstrated approximately 50% reduction in p-STAT3 expression 24 hours after the first dose of WP1066, and at all dose levels. Together these data confirm that WP1066 is effective in suppressing systemic STAT3 activity. Although subsequent dosing 7 days after the first dose given showed similar decreases in p-STAT3, we did not observe a prolonged or cumulative effect, as PBMC p-STAT3 levels had reverted to baseline before the second week of drug administration. This was the anticipated result given that PBMCs are being continuously replenished between the Monday-Wednesday-Friday weekly dosing intervals. One limitation of our study is that we were not able to correlate these changes in PBMCs with TME p-STAT3 expression without immediate pre- and post-treatment tumor tissue to analyze. Analysis of PBMCs and tumor tissue before and after treatment with WP1066 is planned for the current adult phase II clinical trial of WP1066 in GBM patients (NCT05879250) and for future pediatric trials.

A second limitation of our study is that we were not able to obtain reliable pharmacokinetic (PK) data for WP1066. The adult phase I trial in GBM patients initially reported PK data for WP1066 using a preliminary assay that failed to accurately estimate available WP1066 (45). This is in part a result of the unique properties of WP1066, a Michael acceptor that reacts reversibly with nucleophiles, especially sulfhydryl groups of amino acids. WP1066 was formulated to allow for extended release, but it does not follow pharmacokinetics of the naked drug. Despite investigation by multiple laboratories, no method currently exists for measuring free and active forms of WP1066. Although we collected samples for PK analysis in our study, and analyzed the first 3 patients, the results were highly variable, and thus we did not analyze the remaining samples. Further confounding the assay’s accuracy is that PK data are inherently more variable with oral versus intravenous drug dosing. Optimization of the assay is currently ongoing.

Corresponding to the reduction in PBMC p-STAT3 with WP1066 treatment, we observed specific alterations in the proportion and function of peripheral circulating immune cells toward an antitumor immune response, confirming the anti-STAT3 functional activity of WP1066 treatment in this patient population. Indeed, consistent with the preclinical mechanism of action described for WP1066 (52–54), we observed PBMC gene signatures after treatment reflective of increased CD4^+^ and CD8^+^ T cells, increased proinflammatory TNFA signaling, increased inflammatory, stress, and differentiation activity in myeloid cells, and downregulation of Tregs. Notably, while the decreased PBMCs’ p-STAT3 levels reverted to baseline between dosing intervals, there appear to be longer-term downstream effects as evidenced by the immune cell changes observed by RNA-seq analysis at cycle 2, hour 0. However, there did not appear to be a dose-dependent difference between the RNA-seq analyses performed at dose level 2 versus dose level 3. Importantly, these changes were unrelated to concomitant steroid use, as only one patient analyzed was receiving steroids during therapy and the steroid dosage was stable throughout treatment. Whether these peripheral changes reflect shifts in immune cells trafficking to the TME is again unclear without pre- and post-treatment tissue to analyze. However, studies have shown that ablating STAT3 only in murine hematopoietic cells results in marked enhancement of T, NK, and dendritic cell function in tumor-bearing mice and potent local antitumor effects in vivo (28). Thus, it is conceivable that the systemic immune responses induced by WP1066 treatment may be sufficient to generate an antitumor response within the TME, irrespective of the amount of WP1066 that crosses the BBB. Although we observed systemic antitumor immune responses over a short treatment interval, all patients progressed in a relatively short time on therapy. Historically, single-agent immunotherapy, such as immune checkpoint inhibitors, typically requires weeks to months to generate an effective antitumor response (55), and thus it may be unrealistic to expect a significant impact on survival in the context of a phase I study. However, based on our trial results showing both drug activity and possible clinical benefit, further investigation of the efficacy of WP1066 treatment in these patient populations, especially with RT and possibly in the upfront setting for pHGG, is warranted.

## Methods

### Sex as a biological variable.

Our study examined male and female pediatric patients, and no differences in safety profile were detected between the sexes, but only 1 female was enrolled. Thus, the study was not powered to confirm safety profile differences based on sex.

### Study design and participants.

We performed an open-label, single-center, single-arm, 3+3 dose escalation phase I clinical trial design in pediatric patients with primary malignant brain tumors that had progressed or recurred after standard therapy (NCT04334863). The study was conducted at the Aflac Cancer and Blood Disorders Center of Children’s Healthcare of Atlanta under an IRB-approved protocol (AflacST1901). Patients were between the ages of 3 and 25 years, had a Karnofsky or Lansky Performance Scale score of 60 or higher, had previously undergone standard-of-care treatment including surgery, radiation, and/or first-line adjuvant chemotherapy, and had magnetic resonance imaging (MRI) evidence of recurrent or progressive tumor. No single lesion could be larger than 5 cm in maximal diameter. There was no restriction on the number of prior episodes of tumor progression or treatments given. To be eligible for enrollment, a minimum of a 3-week washout was required from the patient’s last dose of myelosuppressive chemotherapy, or 6 weeks from the last dose of nitrosourea-containing chemotherapy. Patients must have received their last dose of an anticancer investigational or biologic drug more than 7 days before enrollment, 21 days before their last antibody therapy, or 42 days before their last immunotherapy. Patients were also required to be more than 12 weeks from autologous stem cell transplantation. Craniospinal radiation must have been completed at least 3 months before enrollment. Other bone marrow irradiation must have been completed at least 6 weeks before and local palliative irradiation at least 2 weeks before enrollment. Patients being treated with high-dose corticosteroids were required to have received a stable or decreasing dose for at least 1 week before enrollment.

A separate cohort of 3 young adult patients with supratentorial H3.3G34R/V-mutant HGG were treated off-study with compassionate-use WP1066 (provided by Moleculin Inc., Houston, Texas, USA) at the University of Michigan C.S. Mott Children’s Hospital, after having received prior treatment with at least standard-of-care radiation therapy and temozolomide chemotherapy. One of these 3 patients was treated with WP1066 and concurrent adjuvant temozolomide after completing standard radiation and had no evidence of disease at the time of WP1066 initiation. While the compassionate-use patients do not contribute to the pediatric phase I data since these data relate to identifying the maximum tolerated dose and describing the safety and tolerability of WP1066 in this population, they are included here because they enrich for the HGG population — in particular, H3G34R tumors that have been shown preclinically to be dependent on STAT3 activity. Thus, data from these patients may provide further information about the preliminary efficacy of WP1066.

### Study treatment.

Eligible patients were assigned to a dose of WP1066 using a 3+3 design algorithm. On-study patients were treated according to the adult HGG regimen with WP1066 administered orally twice daily on Monday, Wednesday, and Friday for the first 14 days in each 28-day treatment cycle. Patients received WP1066 as a powder based on their weight. Immediately before administration, WP1066 was reconstituted into a pharmacy-prepared peppermint-bubblegum-flavored liquid suspension (mixing of the study drug with additional flavored syrups or other liquid additives to improve taste and palatability for children was not allowed because of unknown biocompatibility). Patients could receive WP1066 for up to 1 year (12 cycles) or until disease progression or unacceptable toxicity. The starting dose of WP1066 was 4 mg/kg, which was the tolerated dose in the adult trial of WP1066 at the time of initiation of our pediatric trial with WP1066 (NCT04334863). The planned dosing regimens for investigation were 4, 6, 8, and 16 mg/kg.

Before treatment was advanced to the next dose level, a cohort summary was discussed with the clinical research monitor and approved by a data safety monitoring board. After a baseline MRI, treatment responses were assessed by MRIs every 2–3 months, or earlier if clinically indicated to rule out progression. Laboratory parameters were measured at baseline and before the start of each treatment cycle. Correlative biology blood samples for flow cytometric analysis of PBMC p-STAT3 and PBMC single-cell RNA sequencing for immunophenotyping were obtained before WP1066 treatment and at scheduled time points after the start of treatment. Adverse events were graded according to the National Cancer Institute’s Common Terminology Criteria for Adverse Events, version 5.0.

### Flow cytometry for PBMC p-STAT3 expression.

Flow cytometry for activated p-STAT3 was performed as previously described (45). PBMCs were obtained from patients on cycle 1 day 1 (C1D1) at 0 hours and 4 hours, C1D2 at 0 hours, and C1D8 at 0 hours and 4 hours. Because the p-STAT3 signal is rapidly lost ex vivo, the blood was immediately processed and analyzed. PBMCs were isolated from heparin blood by density gradient centrifugation with Histopaque Ficoll 1077 (Sigma-Aldrich) and counted. Six million cells were then resuspended in 0.6 mL of PBS. Paraformaldehyde (Thermo Fisher Scientific) was pre-warmed at 37°C and added to achieve a final concentration of 2%. The solution was then incubated for 10 minutes at 37°C, chilled on ice for 1 minute, and equally distributed into 5 wells of a 96-well U-bottom plate. For permeabilization, the paraformaldehyde was removed by pelleting of the cells at 700*g* for 5 minutes, resuspension of the cells in pre-chilled 90% methanol, and incubation on ice for 30 minutes. The cells were then pelleted at 700*g* for 5 minutes at 4°C, washed with FACS staining buffer (Thermo Fisher Scientific), and again pelleted at 700*g* for 5 minutes at 4°C. Two wells were then resuspended in staining buffer including the PE-labeled p-STAT3 (Y705) antibody, two wells in staining buffer including isotype control antibody, and one well in staining buffer only. After 1 hour of incubation at room temperature, the cells were washed again with FACS staining buffer before being transferred to FACS tubes for the flow analysis. Duplicate specimens were processed in parallel.

### scRNA-seq.

scRNA-seq was performed on PBMC samples retrieved at C1D1, C1D8, and C2D1 time points. Gene expression libraries were generated and barcoded using the 10x Genomics Chromium Single-Cell 5′ kit and sequenced using the NovaSeq 6000 platform. Cell Ranger v7 was used to align FASTQs to the transcriptome (GRCh38-2020-A), with mode introns=False and Single Cell 5′ PE chemistry options specified. Aligned cell count matrices for each of the 17 samples were merged and analyzed in downstream steps using Seurat v4 functions (56). The dataset was filtered to identify high-quality cells by keeping cells with low mitochondrial content (<10%) and feature counts in the normal range (>300, <2,500). In addition, we filtered out technical doublets using DoubletFinder v2 (57), resulting in 62,000 high-quality cells for further analysis. SCTransform from Seurat was used for the normalization and scaling of counts, and then principal component analysis, neighborhood analysis, clustering, and uniform manifold approximation and projection (UMAP) analysis were performed to identify unsupervised clusters and project the cells to a 2-dimensional embedding for easier interpretation. Upon examining the UMAPs and clustering, we determined there was no batch effect (Supplemental Figure 5), so we did not use the integration and harmonization methods to overcorrect data. SingleR (58) and canonical cell type markers were used to annotate clusters.

### Statistics.

Patient demographic and clinical characteristics were summarized using descriptive statistics. Dose-limiting toxicities were counted according to dose levels, while toxicities within the evaluable patient cohort were categorized and summarized by grade. Progression-free survival and overall survival were analyzed using Kaplan-Meier method. The statistical analyses were performed using R software, version 4.3.0. To identify the proportion of CD4^+^ and CD8^+^ T cells present in the PBMCs, we calculated the module scores (Seurat v4) for all T cells and for different T cell subtype signatures from the literature (59). In addition, gene set enrichment analysis was performed to assess the underlying biological mechanisms active in the PBMCs at different time points. The escape package (60) was used to assess the enrichment of different hallmark gene sets (Molecular Signatures Database [MSigDB]). Wilcoxon’s rank sum tests were used to compare gene expression, module scores, enrichment scores, and cell type proportions between cells or samples from C1D1 versus C1D8 and C1D8 versus C2D1 time points. While we were limited by sample size per time point, we performed a pseudobulk approach when validating the changes in signatures represented in Figures 5 and 6. First, we summed counts for each gene across all the cells in each unique combination of patient and time point (combining replicates). Then, we performed an RNA-seq analysis approach using DESeq2 methodology, including filtering of genes with low counts, estimation of size factors, and normalization. Then, we performed gene set variation analysis (using the GSVA package) treating each patient as an observation within each time point, comparing enrichment scores across time points.

### Study approval.

This study was approved by the Institutional Review Boards of Emory University and Children’s Healthcare of Atlanta before study initiation, and written informed signed consent was obtained from study participants aged 18 years or older or from the legal guardian(s) of study participants less than 18 years of age prior to any study procedures or participation.

### Data availability.

After lockout of the database, all generated data are now available through the Pediatric Cancer Data Commons portal (https://commons.cri.uchicago.edu/pcdc/) and are available in the Supporting Data Values file.

## Author contributions

TJM, ABH, WP, RCC, DA, and RK were responsible for the primary study design. TJM, RCC, and DA conducted the study. HM and MB performed the sequencing and analysis. BY, HR, and KD assisted in flow cytometry analysis. MS provided neuropathology support. ZH performed the clinical data analysis. RCC, TJM, HM, AF, and ZH drafted the manuscript, and ABH, WP, and DA read the draft manuscript for detailed editorial feedback. All authors reviewed and approved the manuscript.

## Funding support

The following entities provided funding support.

- CURE Childhood Cancer (to TJM).
- Peach Bowl Inc. (to TJM).

## Supplementary Material

Supplemental data

ICMJE disclosure forms

Supporting data values

## Figures and Tables

**Table 1:**
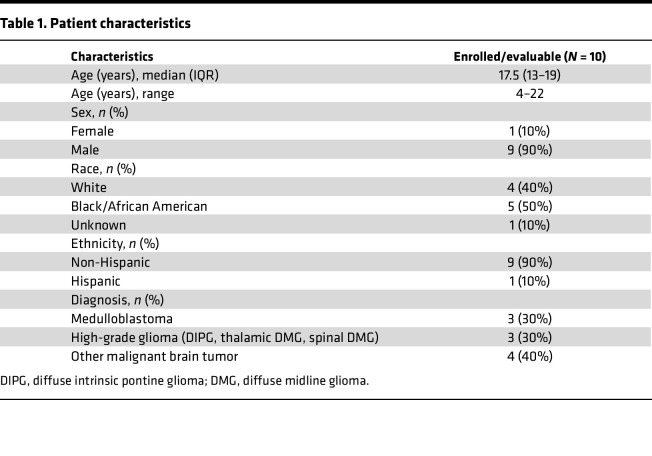
Patient characteristics

**Table 2:**
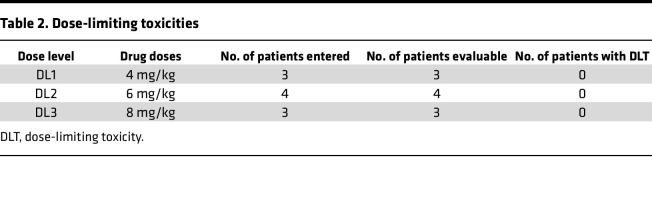
Dose-limiting toxicities

**Table 3:**
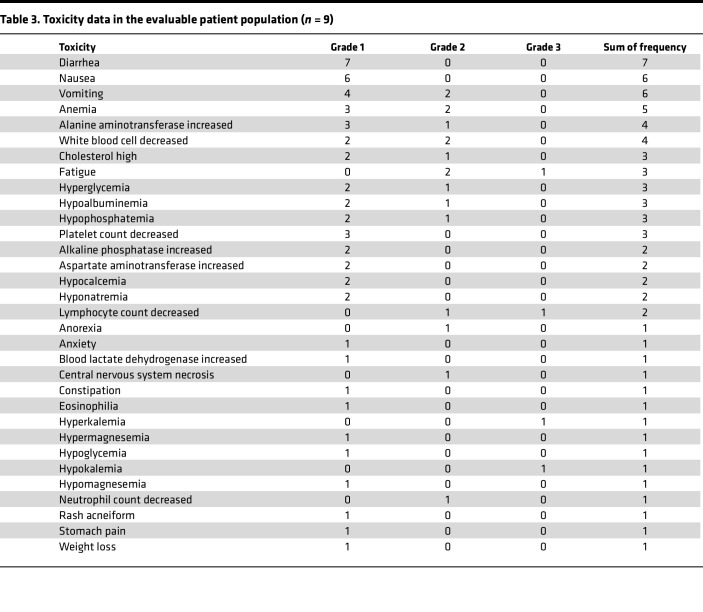
Toxicity data in the evaluable patient population (*n* = 9)

## Version 1 12/11/2025

In-Press Preview

## Version 2 02/09/2026

Electronic publication
