# Supplementary material for: First-in-child phase I trial of p-STAT3 inhibitor WP1066 in pediatric brain tumor patients
Source: JCI Insight. 2025 Dec 11;11(3):e194823. doi: 10.1172/jci.insight.194823 (PMC12893104; doi:10.1172/jci.insight.194823)
Supplement: Supplemental data [file jciinsight-11-194823-s254.pdf]

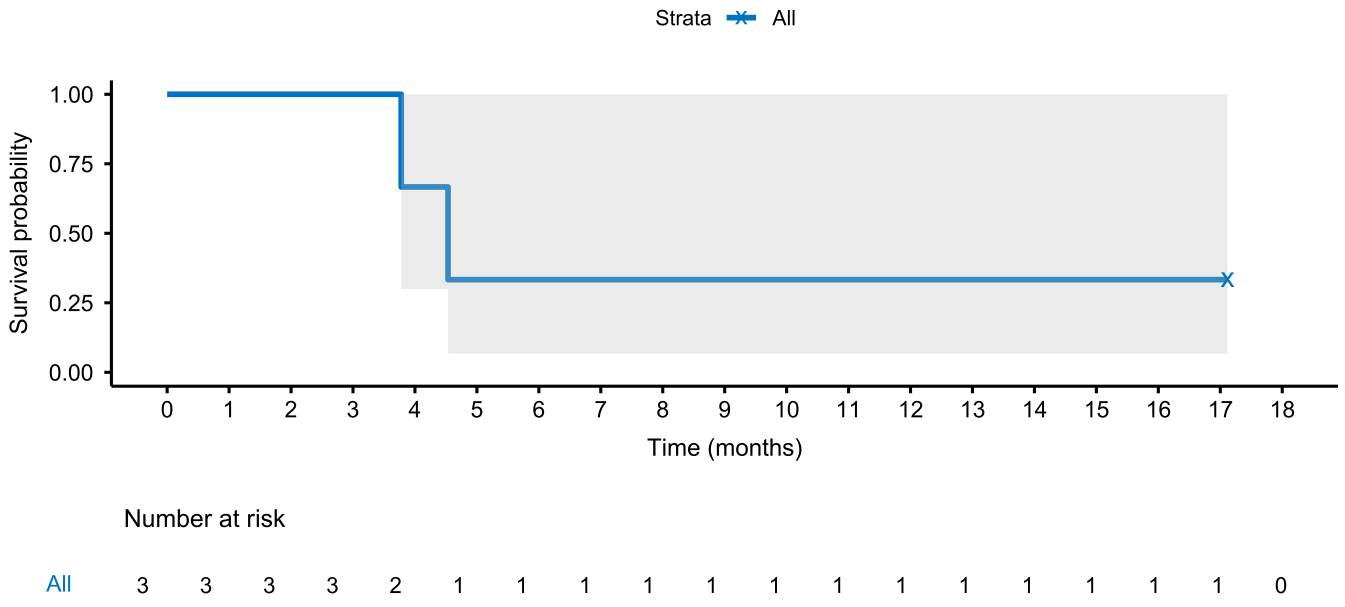

Supplementary Figure 1. Survival of H3G34R/V-mutant high-grade glioma (HGG) patients treated off-study with compassionate use WP1066. Kaplan-Meier plot of the overall survival of three patients with H3G34R/V-mutant HGG who were treated off-study at the University of Michigan with compassionate use WP1066. Note: One patient was alive when they exited the trial and is designated with “x”. The grey area represents 95% confidence interval.

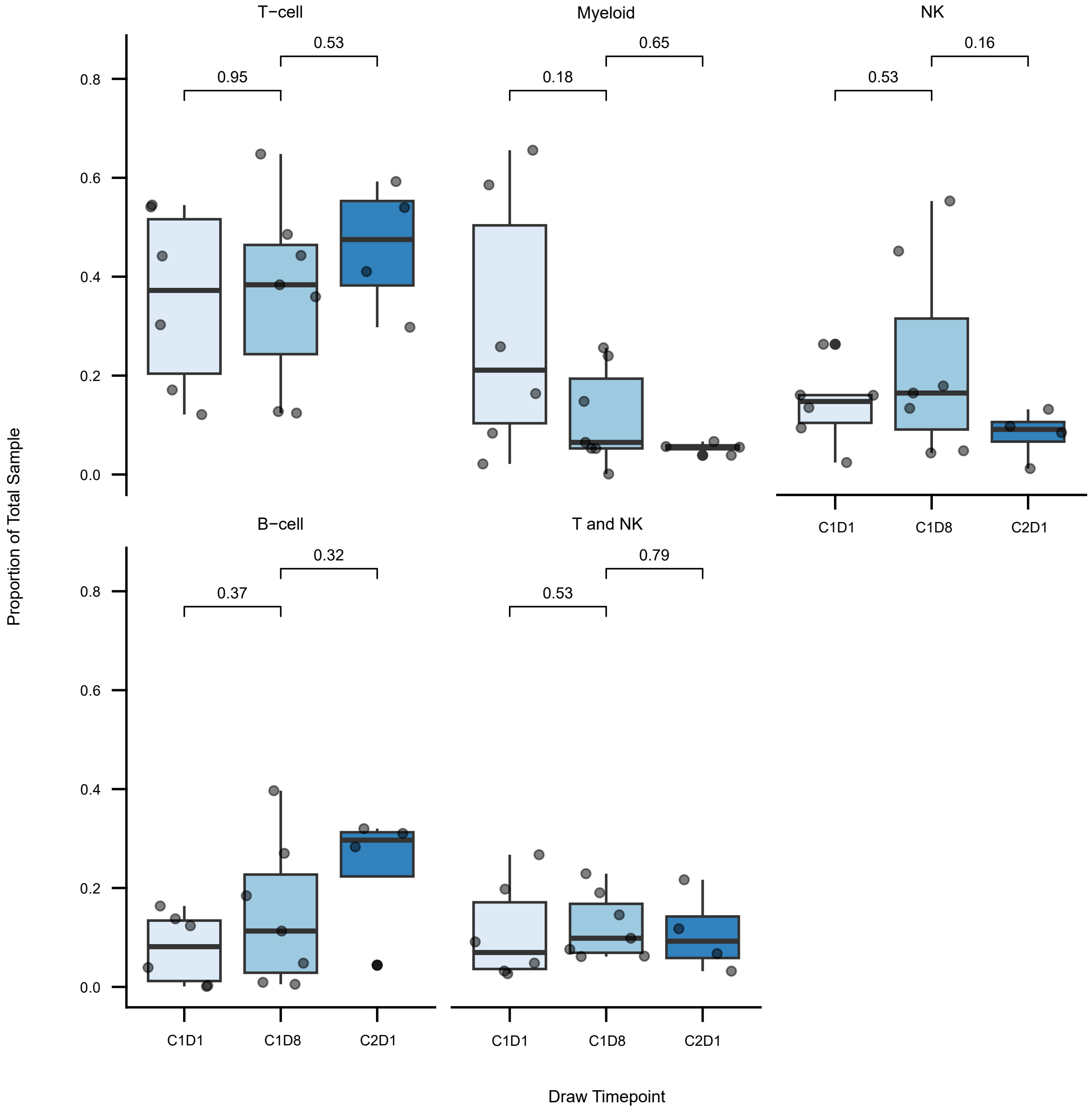

Supplementary Figure 2. Cell type proportions calculated on a sample basis across the timepoints. For each sample, the proportion of cells assigned to each cell type are calculated as the number of cells for that cell type divided by the total number of cells for the sample. These values are shown for each timepoint and Wilcoxon rank sum tests are used to compare the values between timepionts.

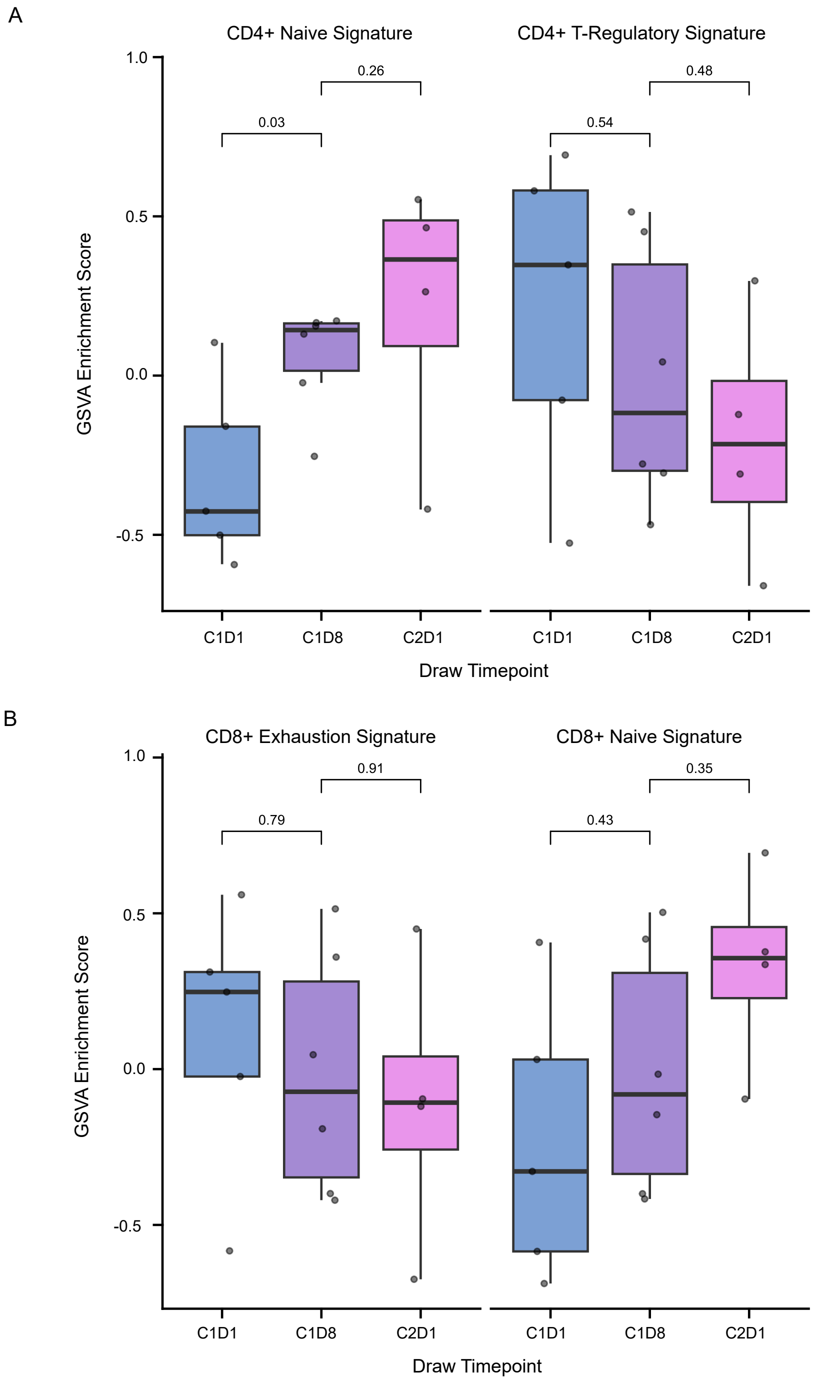

Supplementary Figure 3. Pseudobulk analysis comparing patient-wise changes in T cell subsets in response to WP1066. CD4+ and CD8+ T cell subsets were estimated using phenotype signatures from the literature and assessing the expression of these signatures in T cell subsets in PBMCs. PBMCs analyzed correspond to the PBMC samples analyzed by flow cytometry demonstrating suppressed p-STAT3 after WP1066 treatment (AflacST1901-04, -05, -06, -07, -08) and one additional subject (AflacST1901-010) who did not have flow cytometric analysis for PBMC p-STAT3. Gene set variation analysis was performed on pseudobulk normalized expression values for each sample's CD4+ (A: CD4+ Naïve and CD4+ T-Regulatory signature) and CD8+ (B: CD8+ Exhaustion and CD8+ Naïve signature) subsets. Wilcoxon rank sum tests were used to compare enrichment scores for samples from C1D1 versus C1D8 and C1D8 versus C2D1 timepoints.

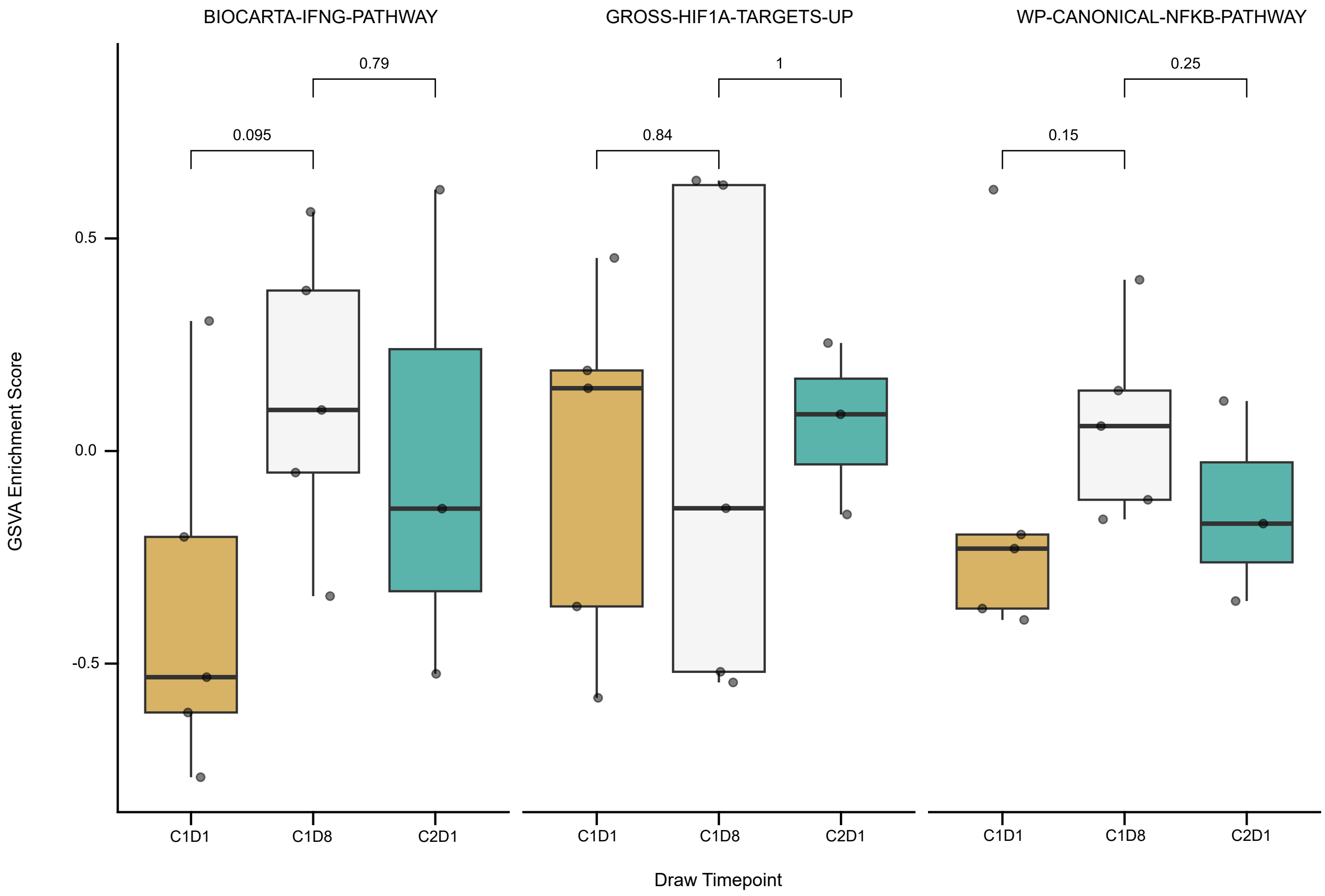

Supplementary Figure 4. Pseudobulk analysis comparing inflammatory pathway enrichment in myeloid cells after WP1066 treatment. Gene set variation analysis was performed on pseudobulk normalized expression values for each sample's myeloid cells to calculate the enrichment of the inflammatory pathway gene set in myeloid cells across time points. Gene sets were obtained from the MSigDb collection. Wilcoxon rank sum tests were used to compare enrichment scores among samples from C1D1 versus C1D8 and C1D8 versus C2D1 timepoints.

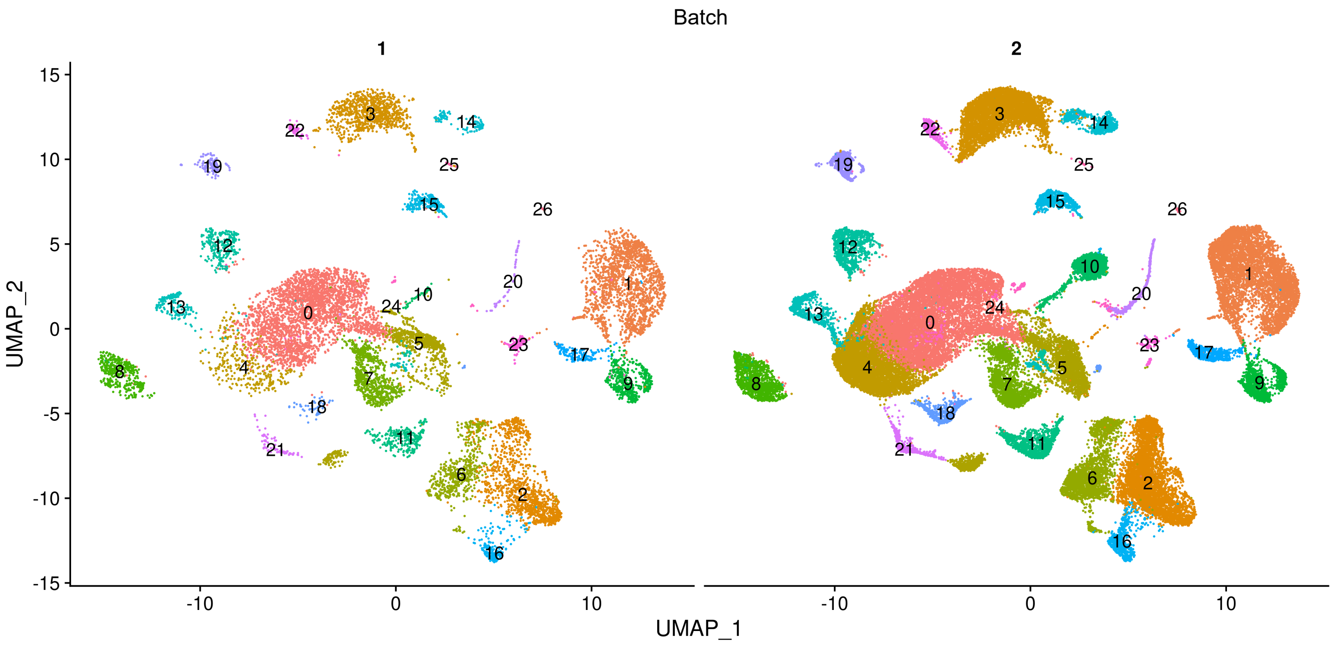

Supplementary Figure 5. UMAP reduction of PBMCs for each batch of samples. After preprocessing, uniform manifold approximation projection plots are generated to show the clustering representation across the two batches of samples sequenced. These UMAPs show there is no batch effect present, with all clusters represented in both batches.
